# Not so bad: avoidance and aversive discounting modulate threat appraisal in anterior cingulate and medial prefrontal cortex

**DOI:** 10.3389/fnbeh.2015.00142

**Published:** 2015-06-10

**Authors:** Michael W. Schlund, Adam T. Brewer, David M. Richman, Sandy K. Magee, Simon Dymond

**Affiliations:** ^1^Barrett Translational Behavioral and Neurobehavioral Laboratory, Department of Behavior Analysis, University of North TexasDenton, TX, USA; ^2^Department of Psychology and Liberal Arts, Florida Institute of TechnologyMelbourne, FL, USA; ^3^Department of Educational Psychology and Leadership, Texas Tech UniversityLubbock, TX, USA; ^4^Experimental Psychopathology Laboratory, Department of Psychology, Swansea UniversitySwansea, UK

**Keywords:** avoidance, threat, fear, anterior cingulate, medial prefrontal cortex, loss discounting, anxiety, neuroimaging

## Abstract

The dorsal anterior cingulate (adACC) and dorsal medial prefrontal cortex (dmPFC) play a central role in the discrimination and appraisal of threatening stimuli. Yet, little is known about what specific features of threatening situations recruit these regions and how avoidance may modulate appraisal and activation through prevention of aversive events. In this investigation, 30 healthy adults underwent functional neuroimaging while completing an avoidance task in which responses to an Avoidable CS+ threat prevented delivery of an aversive stimulus, but not to an Unavoidable CS+ threat. Extinction testing was also completed where CSs were presented without aversive stimulus delivery and an opportunity to avoid. The Avoidable CS+ relative to the Unavoidable CS+ was associated with reductions in ratings of negative valence, fear, and US expectancy and activation. Greater regional activation was consistently observed to the Unavoidable CS+ during avoidance, which declined during extinction. Individuals exhibiting greater aversive discounting—that is, those more avoidant of immediate monetary loss compared to a larger delayed loss—also displayed greater activation to the Unavoidable CS+, highlighting aversive discounting as a significant individual difference variable. These are the first results linking adACC/dmPFC reactivity to avoidance-based reductions of aversive events and modulation of activation by individual differences in aversive discounting.

## Introduction

Discriminating and appraising situations as threatening or non-threatening is important for adaptive approach-avoidance decision-making. Equally important is rapidly and flexibly altering appraisals and associated negative emotional responses following actions that successfully change stimuli/contexts from threats to non-threats. Numerous nonhuman and human investigations on instructed and conditioned fear (for reviews, see Sehlmeyer et al., 2009; Mechias et al., 2010) and anticipatory anxiety and aversion (Straube et al., 2007) highlight central roles for the dorsal anterior cingulate (adACC) and dorsal medial prefrontal cortex (dmPFC) in threat appraisal and fear expression (Milad et al., 2007; Rushworth et al., 2007; Etkin et al., 2011; Shackman et al., 2011; Bravo-Rivera et al., 2014; Kalisch and Gerlicher, 2014). However, a critical gap in our knowledge base concerns what variables and characteristics of threatening situations recruit regions (Rushworth et al., 2007; Etkin et al., 2011; Shackman et al., 2011; Kalisch and Gerlicher, 2014). Accordingly, this investigation employed functional magnetic resonance imaging (fMRI) to examine the effects of avoidance behavior, a prominent emotional coping strategy and core feature of anxiety (Dymond and Roche, 2009; Aldao et al., 2010), as well as trauma and stress related disorders (American Psychiatric Association, 2013), and extinction on threat appraisal and regional activation. Findings obtained will contribute to contemporary theories of adACC/dmPFC function and development of an empirically grounded model of the endophenotypic expressions of pathological avoidance in anxiety which is fundamental to advancing our understanding its etiology, correlates, and prevention.

Anxiety disorders are characterized by exaggerated negative emotional responses to threat, and chronic, ritualized forms of cognitive and behavioral avoidance (Craske et al., 2009). Theories of avoidance highlight central roles for Pavlovian and instrumental learning processes in identifying and coping with threat. During Pavlovian learning, a neutral cue that predicts an aversive unconditioned stimulus (US) will become a conditioned stimulus (CS+) capable of eliciting a conditioned response (CR), while another cue (CS−) does not. Avoidance is then negatively reinforced via instrumental conditioning when it removes the fear-eliciting CS+ threat and subsequently prevents US delivery. In classic two-factor theory, fear and avoidance are closely associated such that CS+ termination and fear reduction are the assumed mechanisms driving and maintaining avoidance (Mowrer, 1947; Bolles, 1973). Instrumental based accounts underscore reduction in the relative frequency of US contact as the key mechanism maintaining avoidance (Herrnstein and Hineline, 1966; Dymond and Roche, 2009). Alternatively, cognitive expectancy theory suggests CS > US expectancies acquired through Pavlovian learning and CS > noUS expectancies acquired during avoidance learning may maintain active avoidance (Lovibond, 2006).

Several contemporary theoretical perspectives highlight a role for adACC/dmPFC in regulating threat appraisal and fear expression based on fear conditioning studies showing greater regional responses to a CS+ threat relative to a CS− (Rushworth et al., 2007; Etkin et al., 2011; Shackman et al., 2011; Kalisch and Gerlicher, 2014). These views may also be extended to human and nonhuman investigations on avoidance that show adACC/dmPFC recruitment to CS+ threats that prompt avoidance (Jensen et al., 2003; Kim et al., 2006; Mobbs et al., 2007, 2009; Delgado et al., 2009; Schlund et al., 2010, 2011, 2013; Bravo-Rivera et al., 2014). However, our current knowledge of relations between adACC/dmPFC and avoidance is limited because most human neuroimaging studies employ avoidance paradigms that restrict imaging analyses to activation associated with both CS+ onset *and* decision to avoid. Using a different approach, Schlund et al. (2013) found that when a CS+ was repeatedly presented during a 16 s threat period and avoidance successfully prevented US deliveries, analyses focusing on temporal dynamics showed CS+ activation initially increased, but then decreased during the threat period even though avoidance responding continued. These findings revealed that adACC activation is not necessarily sustained during avoidance, but instead shows an experience-dependent change when avoidance successfully prevented US deliveries. Regression analysis also revealed that the magnitude of adACC activation was negatively correlated with total avoidance responses. Importantly, the experience-dependent change was observed when avoidance was well-learned, thereby eliminating trial and error learning as an explanation. Such findings suggest adACC/dmPFC can flexibly regulate threat appraisal and fear expression based on avoidance-based local changes in the likelihood of experiencing an aversive outcome. However, the significance of findings is somewhat limited because CS+s associated with *unsuccessful* avoidance were not employed as negative controls.

Results suggesting adACC/dmPFC is sensitive to local reductions in US probability through avoidance seems reasonable given the dynamic relationship between avoidance and the CS > US association. More specifically, it is plausible to suggest that one consequence of successful avoidance is that it transforms the CS+ into a safety-like CS− cue by preventing US delivery. Thus, successful avoidance adds to the CS+ an additional inhibitory association (CS+ > noUS) that coexists with the original excitatory association (CS+ > US) established through prior Pavlovian pairings (see Craske et al., 2014). Another consequence of avoidance is it produces an immediate local reduction in US probability, the net effect of which is a fundamental change in the reinforcement history and associated CS+ threat value, with the duration and extent of change entirely dependent upon the participant's avoidance behavior. In some ways, successful avoidance models extinction which involves learning an inhibitory association to the CS+ and in turn alters the CS+ reinforcement history. This view is consistent with human and non-human investigations of avoidance that report reductions in cognitive US expectancies and physiological fear to CS+s (Starr and Mineka, 1977; Lovibond et al., 2007; Dymond et al., 2011, 2012).

The primary aim of this investigation was to further our understanding of how changes in the CS+ > US association through avoidance modulates adACC/dmPFC responses and subjective ratings of US expectancies, stimulus valence, and fear. Our specific question was to what extent adACC/dmPFC responses are differentially controlled by the prevailing excitatory CS+ > US association verses the temporary inhibitory CS+ > noUS association governed by successful avoidance. This question speaks to the adaptive ability to flexibly alter appraisals and associated negative emotional responses following actions that effectively change stimuli/contexts from threats to non-threats. Evidence showing adACC/dmPFC activation to a CS+ associated with successful avoidance (i.e., prevents US delivery) would suggest control by the prevailing excitatory CS+ > US association and adACC/dmPFC insensitivity to local reductions in US probability. Alternatively, evidence showing the absence of adACC/dmPFC activation to a CS+ associated with successful avoidance would highlight control by the inhibitory CS+ > noUS association and adACC/dmPFC sensitivity to local changes in US probability. The latter finding would help bridge views emphasizing adACC/dmPFC in regulating threat appraisal and fear expression (Etkin et al., 2011; Shackman et al., 2011; Kalisch and Gerlicher, 2014) with views that regional responses reflect an extended choice-outcome history with response dependent positive and negative outcomes (i.e., CS+ reinforcement history) (Rushworth et al., 2007).

The secondary aim of this investigation was to bring a clinically-relevant individual-differences approach to advancing our understanding of relations between adACC/dmPFC function and human avoidance. One important gap in our knowledge concerns how vulnerability factors implicated in the pathogenesis of chronic avoidance coping modulate human avoidance neurocircuitry (Schlund et al., 2011, 2013). Emerging evidence on discounting of rewards and aversive outcomes highlight discounting as a candidate individual difference variable in psychopathology (Rounds et al., 2007; Bickel et al., 2009; Salters-Pedneault and Diller, 2013; Tanaka et al., 2014). In research on anxiety, for example, Salters-Pedneault and Diller (2013) used a behavioral delay discounting task where participants made choices between electric shocks delivered immediately vs. shocks delivered after various time delays and found that increased anxiety and experiential avoidance scores were associated with avoidance of immediate shocks (see also Deluty, 1978). Evidence from human neuroimaging studies examining discounting of gains and losses implicate the anterior cingulate, striatum, posterior cingulate, and lateral prefrontal cortex (Bickel et al., 2009) and have highlighted regional differences in the magnitude of activation to losses. For example, Xu et al. (2009) reported choices involving losses were associated with greater activation in posterior parietal areas, insula, thalamus, and dorsal striatum and choices involving immediate losses differentially activated anterior cingulate cortex, insula, and superior frontal gyrus. Similarly, Tanaka et al. (2014) examined the neural correlates of gain and loss asymmetry (i.e., the “sign effect”) and found the sign effect was associated with a greater insular response to the magnitude of loss than gain and a greater striatal response to the delay of loss than gain. Collectively, more immediate losses are perceived as more aversive or threatening and choice patterns recruit brain regions implicated in threat appraisal and fear expression. Here, we sought to characterize the relation between discounting of delayed losses and adACC/dmPFC activation to CS+ threat to evaluate aversive discounting as candidate individual difference variable. We hypothesized that individuals exhibiting greater aversive discounting in the form of greater avoidance of immediate losses would display greater adACC/dmPFC activation to a CS+ threat.

Using a within-subjects design, we coupled fMRI with a novel delayed avoidance task to examine the effects of avoidance and extinction on threat appraisal and adACC/dmPFC regional activation in healthy adults. The delayed avoidance task was developed to temporally separate CS presentation from avoidance responses to better isolate regional activation to CSs. Prior to neuroimaging, our participants underwent threat conditioning in which two visual CS+ threats predicted US delivery and a safe CS− predicted its absence. Afterwards, participants learned through trial and error they could avoid the US associated with one CS+ threat (Avoidable CS+) but not a second CS+ threat (Unavoidable CS+). Pretraining established CSs as threats and eliminated learning related changes in activation from analyses. Neuroimaging occurred during the delayed avoidance task with CSs presented randomly. Within the same session, extinction testing was performed in which the US was withheld and CSs presented without an opportunity to avoid. We hypothesized the Unavoidable CS+ threat would be associated with greater regional activation and greater ratings of negative valence, fear, and US expectancy compared to both the Avoidable CS+ and Safe CS−.

## Materials and methods

### Participants

Thirty, right-handed adults (*M*_age_ = 24.1, *SD* = 4.3, 16 males) without any reported clinical disorders, metal in the body, or use of medications altering central nervous system functioning and/or pregnancy provided written informed consent. Participants were compensated with a fixed amount for participation and could earn money during the experimental tasks. The Institutional Review Boards for the Protection of Human Subjects at the University of North Texas and Texas Tech University approved this investigation.

### Conditioned stimuli

**Figure 1:**
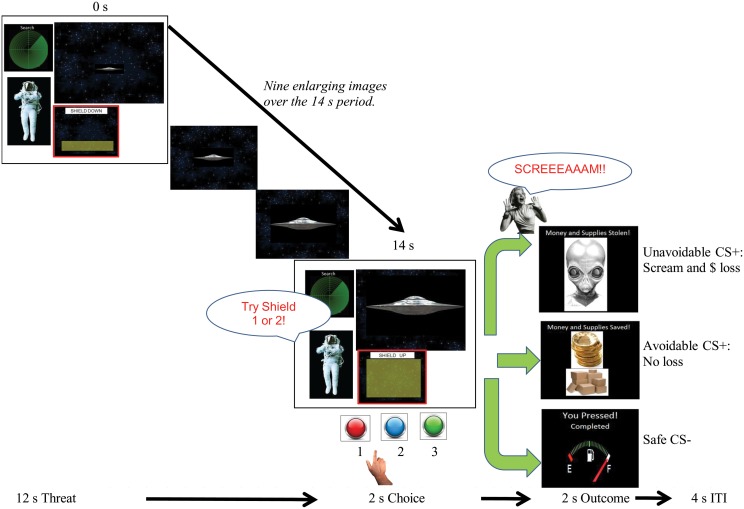
**Schematic and timing of the delayed avoidance task**. Trials lasted 20 s and consisted of a 12 s threat phase during which one of three different CS spaceships (Unavoidable CS+, Avoidable CS+ or Safe CS−) enlarged over time, a 2 s choice phase, a 2 s US/outcome phase for each CS and a 4 s intertrial interval (ITI). During the avoidance task, subjects made a choice between two shields (buttons #1 or #2) that could prevent the aversive US (outcome). Choosing shield #1 to the Avoidable CS+ prevented the US whereas choosing any shield to the Unavoidable CS+ always produced the aversive US. Subjects pressed button #3 to the Safe CS−. During extinction testing, CSs were presented without the aversive US and the opportunity to avoid (i.e., response prevention).

Three images of spaceships served as CSs (see Figure 1 for an example). The US was a empirically validated compound aversive stimulus consisting of the simultaneous presentation of a $1.00 loss prompt and 600 ms female scream (see Delgado et al., 2006; Lau et al., 2008; Schlund et al., 2010, 2011, 2013; Glenn et al., 2012a,b). One CS+ was arbitrarily designated the “Avoidable CS*+*” threat. For this CS+, participants learned through threat conditioning (see below) it predicted US delivery and through trial and error learning (see below) an avoidance response could prevent US delivery. The second CS+ was designated the “Unavoidable CS+” threat. For this CS+, participants learned through threat conditioning it predicted US delivery and through trial and error learning that no amount of responding could prevent US delivery. Thus, instructions were not used to establish CS+s as threats or direct avoidance responding. Lastly, a “Safe CS−” spaceship was established by not pairing it with US delivery.

### Design

The procedure consisted of completing four consecutive steps prior to neuroimaging: (a) Completion of a discounting task with hypothetical money losses; (b) CS pretesting to ensure CSs were viewed as neutral and responding was undifferentiated; (c) Threat conditioning, which established CS+s as threats by pairing them with the US, and establishing the CS− as safe by pairing it with the absence of the US; (d) Avoidance learning, where US presentations could be prevented to one CS+ but not another. Lastly, neuroimaging occurred while participants completed a delayed avoidance task during one scanning run and extinction testing during a second run.

### fMRI data acquisition

The avoidance task and extinction testing were performed during two separate fMRI scans sensitive to blood oxygen level dependent (BOLD) contrast with a 3T Siemens Magnetom Skyra equipped with a 20 channel head coil. T2^*^-weighted echo-planar images consisted of 41 axial oriented slices with voxels measuring 3.5 mm^3^ (repetition time = 2000 ms, echo time = 20 ms, 90° flip angle, field of view = 221 mm, 64 × 64 matrix, 272 dynamics). To minimize equilibrium effects, the first four EPI volumes for each acquisition were discarded. Additionally a high-resolution T1-weighted image was obtained for anatomical reference (192 sagittal slices, voxels 0.9 mm^3^, repetition time 1900 ms, echo time 2.49 ms, field of view 240 mm).

### Procedure

#### Discounting task

Assessment of aversive discounting was determined using an adjusting amount delay discounting task (Du et al., 2002) with hypothetical monetary losses. The rationale for the task rests on supposition that remote aversive events are less aversive or threatening that proximal ones (e.g., McNaughton and Corr, 2004). As such, responses to delayed losses may provide a novel individual difference measure of threat sensitivity and anxiety (e.g., Salters-Pedneault and Diller, 2013). In the task, participants were asked what they would prefer to lose by way of having to pay an amount of money, with paying reflecting avoidance of a more aversive alternative with a higher threat value. Participants were given repeated choices between paying (a) a large $500 delayed loss (under randomized delay conditions of 0.08,0.50, 1, 3, 5, and 10 years) or (b) a smaller $250 immediate loss. Following choice of the large delayed loss, the amount of the small immediate loss decreased by 50% on the subsequent trial. Following choice of the small immediate loss, the amount of the small immediate loss increased by 50% on the subsequent trial, but never exceeded $500. This adjusting procedure determined the point at which the subjective value of the options was equivalent (indifference point) under each delay condition. Values were the resulting small immediate loss after six trials for each delay condition. Aversive discounting was characterized for each subject using area under the curve (AUC; Myerson et al., 2001). An AUC of 1.0 would highlight consistent choice of the immediate loss, meaning delayed losses had a higher threat value and were avoided. By comparison, an AUC of 0 would highlight consistent choice of the large delayed loss, meaning smaller immediate loss had a higher threat value and were avoided.

#### CS pretesting

Participants viewed each CS spaceship for 10 s and provided a rating in three different categories: negative valence (“How much do you dislike the [CS]?”), fear (“How much do you fear the [CS]?”), and US expectancy (“How much money did you lose to the [CS]?”). Ratings were made using a 9-point scale (1 = *Not at all*, 9 = *A lot*).

#### Threat conditioning

A modified Pavlovian fear conditioning paradigm was utilized to establish two excitatory CS+ > US relations for two CS+ spaceships (i.e., threat cues) and establish a CS− spaceship as a safe cue (see Figure 1). The delayed avoidance task shown in Figure 1 was modified for this purpose. Trials lasted 20 s and consisted of a 12 s threat phase during which one presented CS physically enlarged over time (15–90 mm; 8 mm/s), (the 2 s choice phase shown was omitted), followed by a 2 s outcome phase and 4 s intertrial interval. Participants were given a stipend of $10.00 and instructed to watch and learn which spaceships predicted the US and which did not during the 4 min task. CSs were presented for five trials in a randomized order with equal probability and CS+s were always followed by the US. The CS− was followed by a blank screen. Dependent measures included valence, fear, and US expectancy ratings for each CS. Threat conditioning was considered successful when both CS+s were more disliked and feared than the CS− and US expectancies for CS+s were greater compared to the CS−. Ratings provide evidence of the conscious knowledge of differences in cue-outcome contingencies among CSs and differences in associated negative appraisal processes.

#### Avoidance acquisition

Figure 1 provides a schematic of the 4 min task used to establish avoidance prior to neuroimaging. The goal was to train participants to learn that avoidance could prevent the US following the Avoidable CS+ but not following the Unavoidable CS+, and press response button #3 to the CS−. Thus, pretraining was designed to facilitate learning an inhibitory CS+ > noUS association for the Avoidable CS+. Pretraining also eliminated learning related activation during subsequent neuroimaging. Participants were given a stipend of $10.00 and told their task was to keep aliens from taking their supplies and money, they *may* be able to stop an alien ship by choosing between shield #1 or shield #2 depending on which spaceship was present, and to press #3 to allow a “Friendly ship” [CS−] to refuel. Trials lasted 20 s and consisted of a 12 s phase during which one CS (Unavoidable CS+, Avoidable CS+ or Friendly CS−) enlarged over time, a 2 s choice phase, a 2 s outcome phase and 4 s intertrial interval. During the choice phase, when a CS+ was presented participants were prompted to make a choice between two shields (#1, #2) that *may* prevent the aversive stimulus. Choosing shield #1 after the Avoidable CS+ prevented the US and any other response produced US. Therefore, avoidance was acquired through trial and error learning. Regardless of the shield chosen, the Unavoidable CS+ was always followed by the US. Dependent measures included button/shield choice and reaction time (RT) for each CS. Acquisition ended when successful avoidance to the Avoidable CS+ was >80% correct during a 5 trial block (generally two blocks were required). All participants were required to meet criterion before proceeding to neuroimaging.

#### Neuroimaging

Two, 9 min consecutive imaging scans were completed, separated by a ~3 min break. Participants were given a button box with three buttons arranged vertically and described as #1, #2, and #3. Responses were made with the right thumb. During the first scan, the delayed avoidance task was presented (see Figure 1). Here, participants were given a $13.00 stipend and told their task was (again) to keep aliens from taking their supplies by applying what they learned during training. CS order was randomized in blocks of 3 trials and 10 blocks were presented. Dependent measures included button choice and RT for each CS along with valence, fear, and US expectancy ratings obtained at task completion.

During the second scan, extinction testing with response prevention was completed. The extinction testing/task was the delayed avoidance task modified to exclude any opportunity to respond and with all US deliveries withheld. Instructions stated that the shields (buttons) were inoperable, so no avoidance was possible; however, participants still held the button box in the scanner. Participants were not informed about US omission. CS order was randomized in blocks of three trials and 10 blocks were presented. Dependent measures included button presses to each CS (none occurred or were predicted) and valence, fear, and US expectancy ratings obtained at task completion.

### Analyses

#### Neuroimaging

Neuroimaging data analyses were performed using SPM 8 (Wellcome Department of Cognitive Neurology, London UK, http://www.fil.ion.ucl.ac.uk/). Preprocessing procedures included reorientation, slice acquisition time correction, coregistration, within-subject realignment, spatial normalization to the standard Montreal Neurological Institute EPI template with resampling to 2 × 2 × 2 mm voxel sizes, and spatial smoothing using a Gaussian kernel (6 mm full width at half-maximum). High pass filtering was applied to the time series of EPI images to remove any low frequency drift in EPI signal. Head motion was restricted to <3.0 mm in any dimension using the first acquisition as a reference. No participants were excluded.

At the first level, individual subject time series data were analyzed using a multiple regression model. Events of interest modeled included an Unavoidable CS+, Avoidable CS+ and a Safe CS− which served as baseline for sensory and motor responses and absence of US delivery. Only trials with correct responses were used in the analysis. CS+ specific activation was highlighted by creating Unavoidable CS+ > CS− and Avoidable CS+ > CS− contrast images. The effects of avoidance during the delayed avoidance task and during extinction testing was assessed by highlighting differences between CS+s with the contrast [(Unavoidable CS+ > CS−) – (Avoidable CS+ > CS−)]. Localization of activation during the 12 s threat period was revealed by convolving a 12 s boxcar function to the time series which produced a parameter estimate reflecting the magnitude of activation. Additionally, a secondary time course verification of 12 s CS presentations was completed with a supplemental analysis involving a Finite Impulse Response (FIR) model with a 2 s sampling rate. The FIR analysis generated parameter estimates for each voxel every 2 s over the 12 s CSs duration. Participant-specific head movement parameters were also modeled as covariates of no interest.

Functional imaging analyses proceeded through three stages: anatomically restricted localization of sustained activation to the 12 s CSs, time course verification of sustained activation and correlation of brain activation with a measure of aversive delay discounting. First level individual contrast images were carried to a second level for group analyses. Because our a priori hypotheses focused on the anterior cingulate and anterior, medial and ventral frontal regions, a regions-of-interest (ROIs) mask was created using the Automated Anatomic Labeling atlas (AAL; Tzourio-Mazoyer et al., 2002) of the WFU Pickatlas toolbox (Maldjian et al., 2003). Consequently, analyses were restricted to these regions and employed SPMs small volume correction function. Activation for the Unavoidable CS+ and the Avoidable CS+ was separated evaluated relative to the Safe CS− with one-sample *t*-tests thresholded at *p* < 0.005 uncorrected and 20 contiguous voxels. However, no significant differences were found for the Avoidable CS+. The effect of successful avoidance on CS activation during avoidance and extinction was highlighted using the contrast [(Unavoidable CS+ > CS−) – (Avoidable CS+ > CS−)] and one-sample *t*-tests thresholded at *p* < 0.005 uncorrected and 20 contiguous voxels. While these thresholds balance concerns of Type I and Type II error (Lieberman and Cunningham, 2009), all clusters reported during avoidance exceeded a cluster level family-wise error (FWE) correction set at *p* < 0.05. Lastly, multiple regression examined relations between regional activation identified with the Unavoidable CS+ > CS− contrast (via inclusive masking) and AUC discounting measures using the thresholds *p* < 0.01 uncorrected and 20 contiguous voxels. Parameter estimates and contrast values plotted are from significant peak voxels. The location of voxels with significant activation was summarized by their local maxima separated by at least 8 mm, and by converting the maxima coordinates from MNI to Talairach coordinate space using conventional transformations implemented in GingerALE 2.0 (http://www.brainmap.org/ale/). MNI with coordinates are reported and regions assigned neuroanatomic labels using Talairach atlas for guidance. Statistical parametric maps displayed were overlaid onto a reference brain using MRIcron (http://www.sph.sc.edu/comd/rorden/mricron/).

#### Behavioral

For each condition (except Avoidance Acquisition), differences among CS ratings were evaluated via three planned comparisons performed using paired *t*-tests and a criterion alpha set at *p* < 0.05/3, Bonferroni corrected. During neuroimaging of the delayed avoidance task, three planned comparisons were performed to evaluate differences among choice distributions and reaction times using paired *t*-tests and a criterion alpha set at *p* < 0.05/3, Bonferroni corrected.

## Results

### Behavioral performance

**Figure 2:**
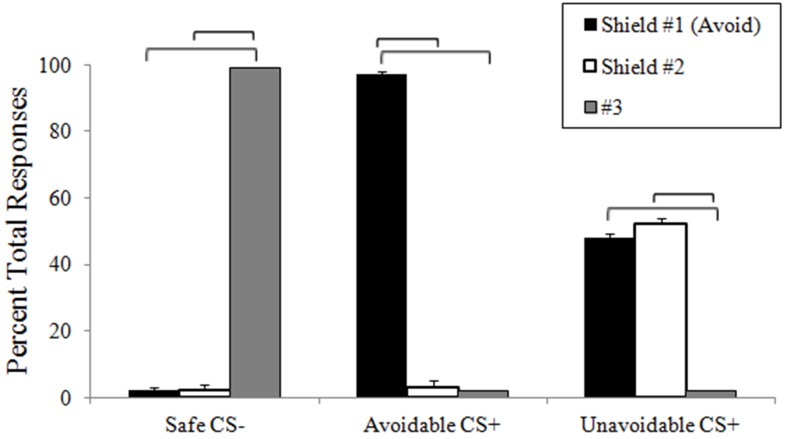
**Response accuracy during avoidance task**. The plot shows the distribution of choices among available responses (buttons 1, 2, and 3) to each CS during neuroimaging. Buttons 1 and 2 were described as “shields” that could help avoid alien attacks. The plot shows subjects consistently (and correctly) choice #3 to the CS− and correctly choice #1 to the Avoidable CS+, which prevented US delivery. In contrast, choices were distributed between #1 and #2 to the Unavoidable CS+ as subjects tried and failed to avoid the aversive US. (Brackets highlight significant differences, *p* < 0.05 corrected. Bars represent 95% confidence intervals).

During the delayed avoidance task, participants chose shield #1 significantly more often to the Avoidable CS+ threat (*M* = 97%, *SD* = 6.4%), which successfully prevented US delivery, and chose button #3 significantly more often for the Safe CS− (*M* = 99%, *SD* = 2.7%), consistent with instructions (Figure 2). No significant differences were found between choices of shields #1 (*M* = 48%, *SD* = 6.6 %) and #2 (*M* = 52%, *SD* = 6.8%) to the Unavoidable CS+ threat, highlighting variable responding as participants tried and failed to prevent US delivery. No significant differences were found among RTs when choosing to avoid the Avoidable CS+ threat (*M* = 576 ms, *SD* = 122 ms) or the Unavoidable CS+ threat (*M* = 562 ms, *SD* = 161) or responding to the Safe CS− (#3 *M* = 563 ms, *SD* = 195 ms). Significant RT differences were not expected given the lengthy 12 s threat period that preceded choices.

### Ratings

**Figure 3:**
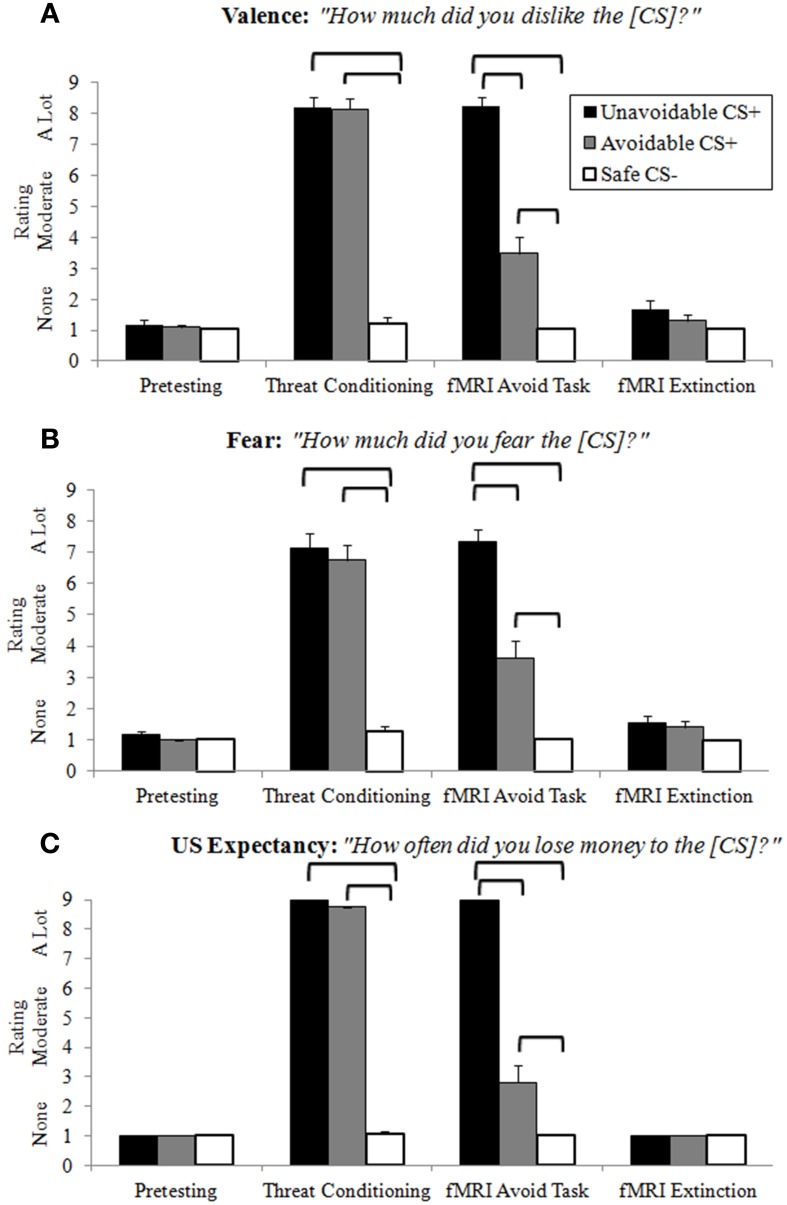
**Retrospective CS ratings**. Following each experimental condition, subjects rated how much they **(A)** disliked (negative valence), **(B)** feared, and **(C)** lost money (US expectancy) for each CS. No differences among CSs were observed after pretesting or extinction. Prior to neuroimaging, threat conditioning paired CS+s with the aversive US which produced significantly higher ratings for CS+s compared to the CS−, indicative of successful differential threat conditioning. For the delayed avoidance task, CS+s were rated significantly higher than the CS− and the Avoidable CS+ threat was rated significantly lower compared to the Unavoidable CS+, demonstrating that successful avoidance reduced threat appraisal. (Brackets highlight significant differences, *p* < 0.05 corrected. Bars represent 95% confidence intervals. See Supplemental Table 1 for details).

Ratings of negative valence, fear and US expectancy provided clear evidence of differential threat conditioning, CS+ modulation by successful avoidance, and extinction learning (Figure 3; Supplemental Table 1). Following pretesting and extinction, all ratings in each category were low and no significant differences among CSs present. Threat conditioning produced significantly higher CS+ ratings in each category relative to the Safe CS−, showing both CS+s functioned as threats. Importantly, no significant differences were observed between CS+s, indicating similar threat values. Ratings in each category for CS+s presented during the delayed avoidance task were significantly higher than the CS−, again demonstrating CS+s acted as threats. However, ratings in each category for the Avoidable CS+ were significantly lower compared to the Unavoidable CS+ and significantly higher compared to the CS−, demonstrating that avoidance significantly reduced ratings of valence, fear and US expectancy, but not to CS− levels.

### Neuroimaging

#### Delayed avoidance task-related activation

**Figure 4:**
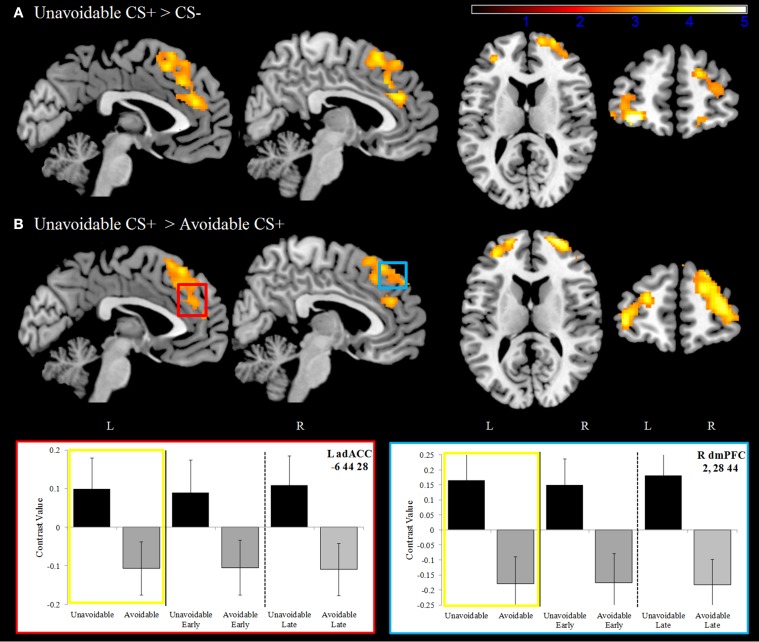
**Dorsal anterior cingulate and dorsal medial prefrontal cortex activation**. **(A)** During the delayed avoidance task (top row), the Unavoidable CS+ was a 12 s threat cue that preceded an unavoidable US delivery. Significantly greater activation was observed to the Unavoidable CS+ relative to the CS− in bilateral dorsal anterior cingulate (adACC) and dorsal medial prefrontal cortex (dmPFC) as well as anterior prefrontal cortex (APFC). **(B)** Activation maps and plots reveal greater activation to the Unavoidable CS+ threat relative to the Avoidable CS+ threat. Plots show contrast values for the session along with early (first half) and late (last half) phases. Yellow boxes correspond to differences appearing in activation maps. The axial slices highlight activation in bilateral APFC, BA10. (Bars reflect 95% confidence intervals).

**Figure 5:**
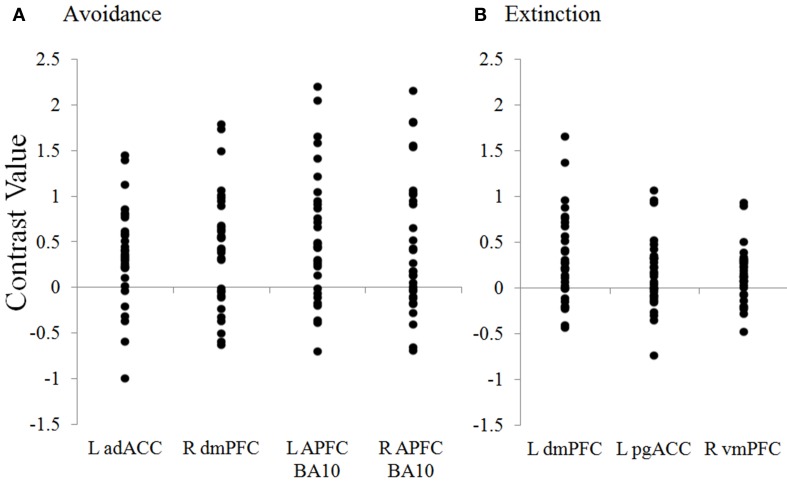
**Distribution of contrast values for regions during avoidance and extinction**. Plots show the distribution of individual subject contrast values for the Unavoidable CS+ > Avoidable CS+ contrast in ROIs during **(A)** Avoidance and **(B)** Extinction. The contrast values plotted reflect the absolute difference between parameter estimates for CS+s. adACC, dorsal anterior cingulate; dmPFC, dorsal medial prefrontal cortex; L APFC, left (R = right) anterior prefrontal cortex; pgACC, pregenual anterior cingulate; vmPFC, ventromedial prefrontal cortex.

**Figure 6:**
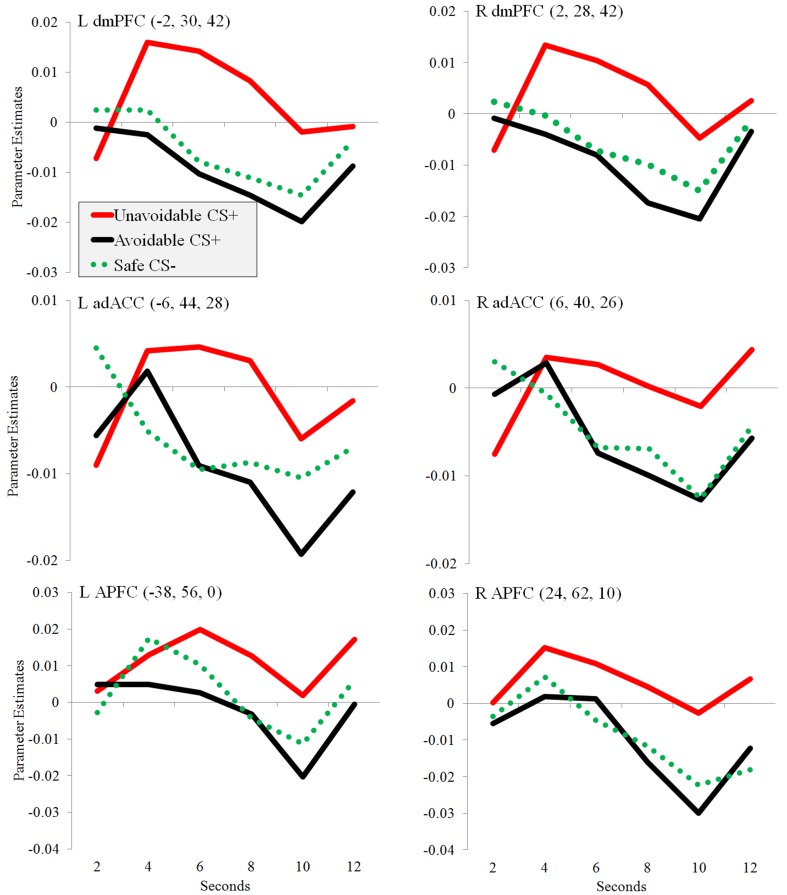
**Changes in regional activation (parameter estimates) during avoidance**. Plots show changes in parameter estimates in ROIs during the presentation of three different 12 s CSs: Unavoidable CS+, Avoidable CS+, and Safe CS−. Activation was observed in bilateral adACC, dmPFC, and APFC.

Significantly greater activation to the Unavoidable CS+ threat relative to the Safe CS− was observed in the adACC and dmPFC (Figure 4A; Table 1), but not for the Avoidable CS+ threat. Similarly, adACC and dmPFC activation was significantly greater to the Unavoidable CS+ threat relative to the Avoidable CS+ threat (Figure 4B; Table 1; see Figure 5 for individual subject contrast values). Plots of contrast values for the session and early and late phases reveal that regional activation was sustained and did not decline during the session. Activation to the Unavoidable CS+ threat also did not decline within the imaging session, highlighting that the Unavoidable CS+ remained a threat and the US remained aversive. Consequently, the reduced activation observed to the Avoidable CS+ cannot be attributed to time or US habituation. Finally, the greater activation to the Unavoidable CS+ threat suggested by results obtained with a 12 s boxcar regressor were verified through FIR time course validation (Figure 6). Plots for adACC, dmPFC, and APFC reveal Unavoidable CS+ activation was sustained during the 12 s threat period while Avoidable CS+ and CS− activation showed markedly similar declines during the 12 s threat period.

**Table 1:** **ROI results for avoidance, extinction, and aversive discounting**.

		**MNI**			**Voxel Z**	**Voxel *p***	**Cluster size**
		**x**	**y**	**z**			
**AVOIDANCE:**							
Unavoidable > Safe CS−	L adACC	−8	42	24	3.6	<0.001	1538
	R adACC	6	40	26	3.46	<0.001	
	R dmPFC	6	34	52	3.46	<0.001	
	L dmPFC	−2	32	54	3.46	<0.001	
Unavoidable > Avoidable	R APFC	24	62	10	4.17	<0.001	1007
	L APFC	−36	58	0	3.73	<0.001	520
	L adACC	−6	44	28	2.93	0.002	650
	R adACC	6	40	25	3.17	0.001	
	R dmPFC	2	28	44	3.11	0.001	
	L dmPFC	−2	30	42	3.1	0.001	
**EXTINCTION**:							
Unavoidable > Avoidable	L dmPFC	−8	44	38	3.06	0.001	41
	L pgACC	−8	48	12	2.81	0.002	27
	R vmPFC	2	38	−18	3.3	<0.001	20
**AVERSIVE DISCOUNTING**							
	L dmPFC	8	44	46	2.74	0.003	697
	L adACC	−8	44	24	3.24	0.001	459
	R adACC	12	38	26	2.88	0.002	60

#### Extinction testing-related activation

**Figure 7:**
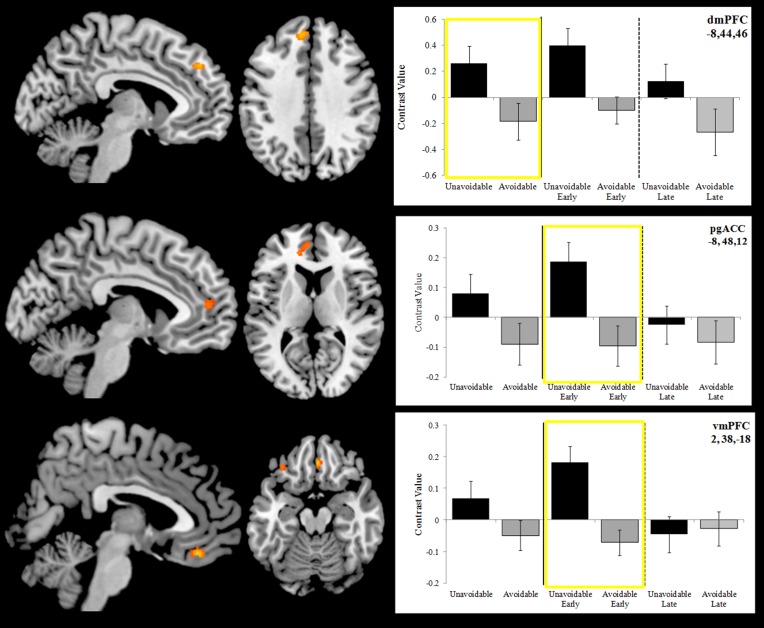
**Differences in activation between the Unavoidable CS+ and Avoidable CS+ during extinction**. Plots show contrast values for the session and early (first half) and late (last half) phases. Values highlighted within yellow boxes correspond to differences appearing in activation maps. The top plot highlights a significant difference in activation in dmPFC for the session. The remaining two plots highlight significant differences in activation in pregenual anterior cingulate (pgACC) and ventromedial prefrontal cortex (vmPFC) for the early phase. (Bars reflect 95% confidence intervals).

Activation to the Unavoidable CS+ threat relative to the Avoidable CS+ threat during the session was restricted to the dmPFC (Figure 7; see Figure 5 for individual subject contrast values). However, analyses of within session changes in activation revealed there was significantly greater activation to the Unavoidable CS+ threat relative to the Avoidable CS+ threat during the early (first half) of the session in pregenual anterior cingulate (pgACC) and ventromedial prefrontal cortex (vmPFC). These within session changes are consistent with changes in threat appraisal that would be predicted to occur under extinction when CSs are presented without the US.

### Brain-behavior relations

**Figure 8:**
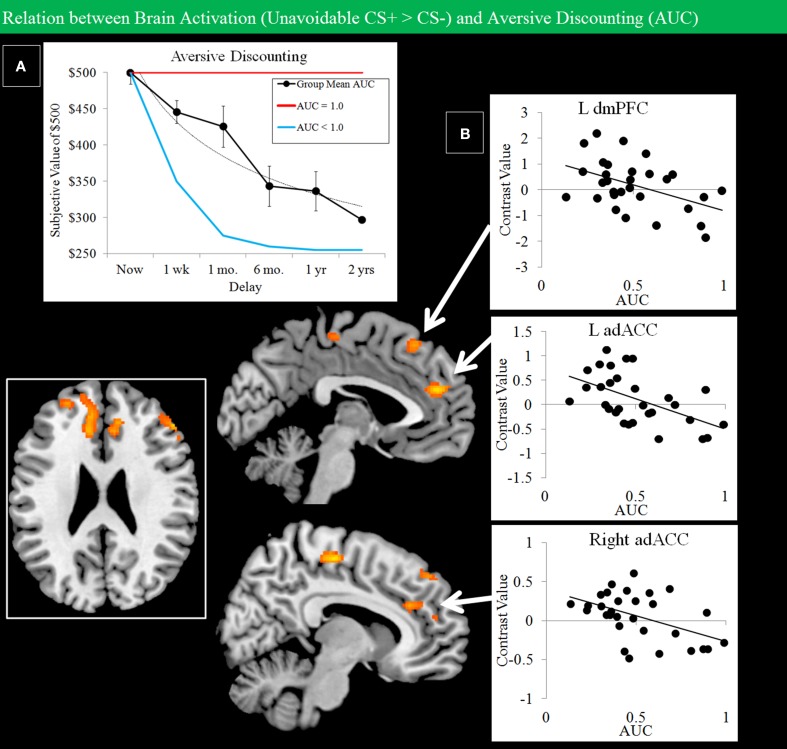
**Negative correlations between Unavoidable CS+ threat activation and aversive discounting**. **(A)** Group data show discounting of hypothetical monetary loss with increasing delay (bars reflect 95% confidence intervals). On the task, choice reflected avoidance of the more aversive option. An AUC of 1.0 (red function: no discounting) highlights choice of an immediate small loss over a large delayed loss. In contrast, an AUC of 0 (blue function: steep discounting) highlights consistent choice of a large delayed loss over a small immediate loss. **(B)** Plots show negative correlations between Unavoidable CS+ activation and AUC in left dmPFC and left/right adACC. Results show subjects with greater aversive discounting (greater avoidance of small immediate loss) showed greater activation the Unavoidable CS+ threat.

Grouped data showed evidence of discounting of losses with increased delay (Figure 8A). A regression analysis constrained to regions showing activation for the Unavoidable CS+ > CS− contrast was used to examine how individual differences in discounting, expressed as AUC, modulated activation. Regional activation and discounting were negatively correlated in dmPFC and bilateral adACC (Figure 8B; Table 1). Therefore, individuals with a lower AUC and who were more avoidant of immediate losses displayed increased activation to threat in adACC and dmPFC.

## Discussion

Using a within-subjects design and fMRI, we examined the effects of avoidance and extinction on threat appraisal and regional activation. Major findings were (a) an Avoidable CS+ threat relative to the Unavoidable CS+ threat was associated with reductions in ratings of negative valence, fear, and US expectancy and reduced regional activation and (b) individuals who exhibited greater aversive discounting and were more avoidant of immediate losses, displayed greater activation to an Unavoidable CS+ threat. Moreover, Unavoidable CS+ activation was sustained or increased during CS presentation and activation was sustained throughout the avoidance task but declined during extinction. These findings suggest adACC/dmPFC supports flexible threat appraisals through sensitivity to avoidance based reductions in the local probability of US delivery. They also bridge views that adACC/dmPFC plays a central role in regulating threat appraisal and fear expression expression (Etkin et al., 2011; Shackman et al., 2011; Kalisch and Gerlicher, 2014) with views that regional responses reflect an extended choice-outcome history with response dependent positive and negative outcomes (Rushworth et al., 2007).

The differential adACC/dmPFC responses observed to Avoidable/Unavoidable CS+s identifies characteristics/variables associated with threatening situations that control regional activation. In particular, our findings identify successful avoidance and associated reductions in US delivery as an important variable mediating threat appraisal and fear expression. The differences in activation observed between the Avoidable CS+ and Unavoidable CS+ parallel results of fear generalization studies that report a reduction in regional activation along the stimulus continuum from CS+ to CS− (Lissek et al., 2014), the effects of controllability of immediate and proximal aversive events and reductions in the negative impact of aversive events (Maier, 2015), studies on anticipatory anxiety showing regional activation during anticipation of phobia-relevant stimuli (Straube et al., 2007) and studies showing how reappraisal of anticipated threat recruits medial and lateral prefrontal regions and reduces anxiety (Yoshimura et al., 2014). Consistent with studies on extinction learning (Phelps et al., 2004; Quirk and Mueller, 2008), we also observed significantly greater pgACC and vmPFC activation to the Unavoidable CS+ threat relative to the Avoidable CS+ threat during the early phase of extinction. During extinction, participants learned an additional CS+ inhibitory association through US omission. The reduced pgACC and vmPFC activation observed to the Avoidable CS+ suggests the inhibitory CS+ > noUS association had been acquired through avoidance before extinction testing, which corresponds with the consequences of avoidance discussed in the Introduction.

These results also contribute to translational research on anxiety pathology. Our approach highlights active avoidance as a potentially useful model for elucidating the brain mechanisms supporting the dynamic relationship between threat appraisal, fear expression and response outcomes that alter threatening situations. Using a novel avoidance paradigm which delayed avoidance responding following CS presentation, we showed avoidance success modulated adACC/dmPFC activation along with ratings of negative valence, fear and US expectancies. Previous investigations on anticipation of aversion have also reported adACC activation along with results highlighting phasic and sustained activation patterns in different brain regions (e.g., Grupe et al., 2013). The reduction in activation we observed to the Avoidable CS+ threat seems quite reasonable in light of the effectiveness of avoidance coping in anxiety disorders. One might even speculate that the decreasing activation we observed during the threat period to the Avoidable CS+ and Safe CS− reflects an active dampening process. Thus, adACC/dmPFC dysfunction in anxiety may manifest as insensitivity to response produced local changes in US probability, an inability to accurately associate long term changes in US probability with a CS+ or an inability to engage an active dampening process to avoidable and non-threatening stimuli.

We found support for aversive discounting as an individual difference variable that may contribute to research on threat and anxiety pathology. Clinical applications of reward discounting have advanced our understanding of dysfunction in various clinical populations, especially in substance abuse (Bickel et al., 2012; for a meta-analysis see MacKillop et al., 2011). Evidence from human neuroimaging research on discounting also suggests different brain mechanisms are involved at different temporal delays (McClure et al., 2004; Ballard and Knutson, 2009). We found individuals who exhibited steeper loss discounting, that is, subjects who were more avoidant of immediate losses, also displayed greater activation to an Unavoidable CS+ threat. These findings support and extend a growing literature investigating relations between aversive discounting, threat appraisal-reactivity and anxiety pathology (Rounds et al., 2007; Salters-Pedneault and Diller, 2013; Tanaka et al., 2014).

The present investigation has potential limitations and the findings raise empirical questions that should be addressed in future studies. First, the CS+ differences observed as a function of successful and unsuccessful avoidance might be enhanced with an aversive US, such as electric shock. Despite the practical and ethical barriers that exist in its application with vulnerable populations such as children, adolescents and those with anxiety disorders (e.g., Britton et al., 2011), it would be salutary to replicate the present findings with a shock US. Second, the inclusion of other independent physiological measures, such as skin-conductance, pupil dilation or fear-potentiated startle responses, would supplement the existing measures of fear conditioning and avoidance. Third, the present paradigm has utility as a translational model of putative neurobehavioral differences in avoidance and threat appraisal in those with and without an anxiety disorder. Finally, an important area for future investigations will be to use parametric designs that manipulate US probability and delays to examine the effects on regional activation and approach-avoidance decision making.

## Conclusions

Altering threat appraisals and associated negative emotional reactions following actions that change situations from threats to non-threats is important for adaptive approach-avoidance decision-making and emotional health. This investigation employed fMRI and healthy adults to examine the effects of avoidance, which is prominent in anxiety, and extinction on threat appraisal and adACC/dmPFC regional activation. Findings were consistent with and extend a number of contemporary theories of adACC/dmPFC function. We concluded that differences in CS+ activation associated with successful avoidance reflect a regional sensitivity to avoidance based reductions in the local US probability. We propose that adACC/dmPFC dysfunction in anxiety may manifest as insensitivity to response produced local changes in US probability, an inability to accurately associate long term changes in US probability with a CS+ or an inability to engage an active dampening process to avoidable and non-threatening stimuli. Another finding with translational value was results showing individuals exhibiting greater aversive discounting—more avoidant of immediate loss compared to a larger delayed loss—also displayed greater activation to the Unavoidable CS+. We concluded that aversive discounting may be a candidate individual difference variable that modulates regional activation to CS+ threat in anxiety pathology. Future investigations are necessary to further elucidate relations between adACC/dmPFC sensitivity and variables, such as delay and US probability, which also influence approach-avoidance decision-making.

### Conflict of interest statement

The authors declare that the research was conducted in the absence of any commercial or financial relationships that could be construed as a potential conflict of interest.
