# Supplementary material for: Not so bad: avoidance and aversive discounting modulate threat appraisal in anterior cingulate and medial prefrontal cortex
Source: Front Behav Neurosci. 2015 Jun 10;9:142. doi: 10.3389/fnbeh.2015.00142 (PMC4461832; doi:10.3389/fnbeh.2015.00142)
Supplement: Supplementary file 1 [file Table1.PDF]

Supplemental Table 1. Mean and standard deviation for CS ratings.

Category Rating	CS	CS pretesting		Threat Conditioning		fMRI Avoidance Task		fMRI Extinction Task	
		M	*SD*	*M*	*SD*	*M*	*SD*	*M*	*SD*
Dislike	Unavoidable CS+	1.19	0.74	*8.19	1.90	*8.23	1.73	1.69	1.51
	Avoidable CS+	1.09	0.39	*8.16	1.93	^*3.48	2.95	1.31	0.95
	Safe CS-	1.03	0.18	1.23	0.96	1.03	0.18	1.03	0.18
Fear	Unavoidable CS+	1.19	0.54	*7.13	2.69	*7.35	2.03	1.55	1.16
	Avoidable CS+	1.00	0.00	*6.74	2.86	^*3.61	3.07	1.41	1.10
	Safe CS-	1.03	0.18	1.26	0.96	1.03	0.18	1.00	0.00
US Expectancy	Unavoidable CS+	1.00	0.00	*9.00	0.00	*9.00	0.00	1.00	0.00
	Avoidable CS+	1.00	0.00	*8.74	1.44	^*2.81	2.18	1.00	0.00
	Safe CS-	1.00	0.00	1.06	0.36	1.03	0.18	1.00	0.00

*CS+ significantly different from CS-

^AvoidableCS+ significantly less than UnavoidableCS+ (all p < .05, corrected)
